# The Cause of Malarial Fever

**Published:** 1880-01

**Authors:** 


### EXCERPTA.

The Cause of Malarial Fever.—I translate the following from an anonymous quotation in a German newspaper:

"On the 1st June last the Professor of Pathological Anatomy in Rome, Tommaisie, submitted to the Academy there the results of the investigations which he and Prof. Klebs, of Prague, at the suggestion of the latter, had made. Last spring boih spent some weeks in the Agro Romano, the modern classic land of swamp fever, examining the lower strata of its atmosphere, its solid component parts, and its stagnant water, and discovered in the first two a microscopic fungus, consisting of numerous movable, shining spores, of a long, oval form, with a diameter of 0.9 millimeters.

"This fungus having been cultivated in different fields of nutrition, and separated by careful filtration from the fluids in which they were collected, was inserted under the skin in dogs. Similar applications were made with the solid microscopic particles (*festen microscopischen partiklen*), obtained by washing large quantities of the surface soil. Fever with a regular typical course attacked the brutes thus treated with free intervals, continuing as long as sixty hours, and the blood rose to a heat of 42° centigrade during the ague fit, the healthy temperature of the dog being from 38° to 39°. The filtered water caused only slight alterations of temperature, even when used in considerable quantity, and the fever caused by it had no intermittent character. The spleen of the dogs sick with the artificial fever had the same acute swelling as that of the man suffering with ague, and besides contained large numbers of the characteristic fungus which was also abundant in various lymphatic glands. Since the fungus grows in a stem-like form they called it the *Bacillus malariæ.* Further particulars will be found in the July number of Klebs' periodical."

So far the translation. Having set forth in the *Pacific Medical and Surgical Journal* for September, 1878, a list of reasons for rejecting the generally received theory that malarial fever is caused by poison in the atmosphere or microscopic fungus, I feel as if it were my place either to fight or surrender to Klebs. It would be unfair to assert that his results and reasonings are fairly represented in the quotation given above; but no other means of knowing his positions are within my reach until I can obtain his periodical. Meantime, let me say that the evidence stated is far from conclusive. We are not told whether these experiments were performed in the malarious district, for if they were, the dogs might have fallen sick without inoculation. Neither is anything said upon the point whether dogs ever take the swamp fever naturally, or, if not, why not? Nor whether the *Bacillus malariæ* is found in the spleen and lymphatic glands of men sick with ague; nor whether that fungus is found in districts free from swamp fever; nor why people in the malarial districts are relatively secure against the attack if they sleep high above the ground or in substantial houses, warmed every night by a fire, or if they sleep on a hill a few hundred feet above the general level of the valley or plain. If Klebs really made a conclusive public demonstration of the truth of his claim, it seems singular that a translation in full has not appeared in the many English journals; for surely, such a discovery would be of vast importance to the human race, and in comparison with it, most of the matters that have filled the columns of the newspapers for the last four months are profoundly insignificant.—*John S. Hittell, in Pacific Med. and Surg. Jour.*

This is a revival of the Salisbury theory, and should not be again too hastily condemned.—[Ed. Atlanta Med. and Surg. Jour.

